# Multi-channel Si-liquid crystal filter with fine tuning capability of individual channels for compensation of fabrication tolerances

**DOI:** 10.1186/1556-276X-7-387

**Published:** 2012-07-12

**Authors:** Anna Baldycheva, Vladimir A Tolmachev, Kevin Berwick, Tatiana S Perova

**Affiliations:** 1 Department of Electronic and Electrical Engineering, Trinity College Dublin, College Green, Dublin 2, Ireland; 2 Ioffe Physical Technical Institute, Polytechnicheskaya 26, St. Petersburg, 194021, Russia; 3 Department of Electronic and Communications Engineering, Dublin Institute of Technology, Kevin St, Dublin 8, Ireland

**Keywords:** Multi-cavity photonic crystal, Coupled Fabry-Pérot resonators, Liquid crystal devices, Integrated optics devices, Wavelength filtering devices, Lithography error, Line-width variation, Critical dimension variation, 42.60.Da, Resonators, Cavities, Amplifiers, Arrays, Rings, 42.60.Fc, Modulation, Tuning, Mode locking, 42.70.Df, Liquid crystals

## Abstract

In this study, a technique for the optimization of the optical characteristics of multi-channel filters after fabrication is proposed. The multi-channel filter under consideration is based on a Si photonic crystal (PhC), tunable liquid crystal and opto-fluidic technologies. By filling air grooves in the one-dimensional, Si-Air PhC with a nematic liquid crystal, an efficiently coupled multi-channel filter can be realised in which a wide stop band is used for channel separation over a wide frequency range. By selectively tuning the refractive index in various coupled cavities, continuous individual tuning of the central channel (or edge channels) up to 25% of the total channel spacing is demonstrated. To our knowledge, this is the first report on the electro-optical solution for the compensation of fabrication tolerances in an integrated platform.

## Background

Although Si fabrication technology has significantly developed over the last 20 years, one of the main problems for optical, nano-scale periodic structures, such as Fabry-Perot interferometers and multi-channel photonic crystal (PhC) filters, remains in defining of the critical dimensions precisely in the system [1-3]. In general, structural deviations and non-uniformities present in the patterned features on the wafer occur for three principal reasons. First of all, there is the fundamental diffraction limit of the projection optics. Secondly, the mask pattern differs from the original design due to limitations in the mask fabrication process. Finally, random and systematic variations inevitably occur in a multitude of lithographic process parameters, such as focus and exposure [4]. Fabrication tolerances for modern e-beam lithography are usually assumed to be a minimum of about 5% to 10% of the nominal target dimensions. This crucially affects the optical characteristics of multi-channel filter devices [5-7]. It changes the precise wavelength position of individual channels; it increases the out-of-band reflection and causes an attenuation of the maximum intensity, resulting in a lowering of the quality factor, *Q*. One of the most promising solutions to these problems may be a filter device with low-power and low-loss capability to compensate for the optical filter deviations. This would allow fine tuning of individual channels in the filter system by varying the temperature or by applying an electric field. However, one of the main challenges in the realisation of tunable multi-channel devices remains the fixed channel spacing or free spectral range which cannot be easily tuned due to the strong coupling between channels. One of the more successful attempts to tackle this problem was presented in [8], where the authors demonstrated a solution for an integrated platform. This approach requires the incorporation of metallic micro-heaters into large 245 μm resonators, demanding precise temperature control of the device during operation.

To our knowledge, there have been no reports in the literature on addressing this problem using electro-tuning for the individual channels in a multi-channel system that is compatible with complementary metal oxide semiconductor technology. The design of the tunable multi-channel filter proposed in this work is based on a one-dimensional (1D) Si photonic crystal (PhC) using opto-fluidics and liquid crystal (LC) technologies [2,5,9-14]. An LC is one of the most attractive tuning material for Si-based integrated devices, enabling tuning of the resonance modes using low applied voltages (from 1.5 V) with negligible absorption during device operation. By creating optical cavities within the periodic structure by infiltration with LC, the PhC mirror can be transformed into a highly efficient coupled multi-channel filter [15]. A coupled multi-cavity PhC system has significant advantages over other types of coupled resonators in terms of device simplicity, ease of integration on a Si chip, and power consumption. We extended the continuous fine-tuning capability of LC single microcavity to a system of individually tunable coupled multi-cavities. The application of an electric field to each cavity was done individually, and its continuous selective variation across all cavities allows to overcome the problems related to the strong coupling between channels. Using an example of a coupled triple-cavity PhC filter operated using the first SBs, we have developed a simple model for easier manipulation of the LC within individual cavities, enabling the independent fine tuning of each channel in the overall system. We note that the model suggested can be extended to a higher number of coupled cavities (defects) and, therefore, to a higher number of resonances, with an improved *Q* value.

## Methods

**Figure 1:**
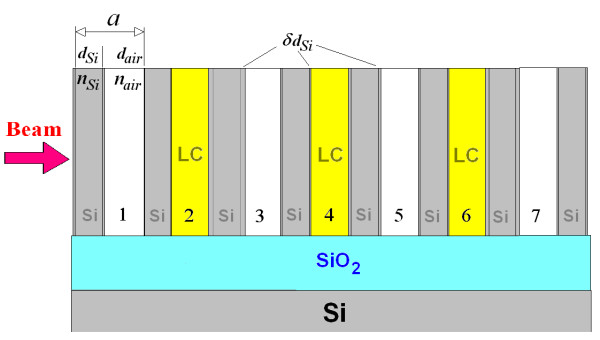
**Schematic diagram of a 7.5 period, 1D, PhC with triple LC defects.** The defects are realised in the second, fourth, and sixth periods (denoted by numbers). A Si wall thickness with fluctuation ± δ *d*_Si_ in each lattice period causes a variation of groove thickness $d_{\text{air}}=a-d_{\text{Si}}\pm \delta d_{\text{Si}}$. The lattice constant is $a=d_{\text{Si}}+d_{\text{air}}$. The optical contrast of the defect-free PhC is *n*_Si_*/n*_air_.

A free-spaced, microstructured, grooved Si structure, with quarter-wavelength Si-Air layers of optical thicknesses $n_{{}_{\text{Si}}}d_{{}_{\text{Si }}}=n_{{}_{\text{air}}}d_{{}_{\text{air}}}=m\lambda_{\text{m}}/4$ (where *m* is the order of the stop bands, SBs), is considered here as a model for an ordinary 1D PhC mirror. The refractive index of air, *n*_air_, is 1, while the refractive index of Si is taken as *n*_Si_ = 3.48. The coupled resonators-induced transparency around the wavelength λ_m_ occurs when optical or geometrical defects are introduced into the perfect periodic structure (Figure 1) [16,17].

The creation of a microcavity resonator requires a doubling of the optical thickness of certain grooves, $n_{\text{def}}^{\left(\text{i}\right)}d_{{}_{\text{air }}}=m\lambda_{\text{m}}/2$, where *i* is the number of the groove. This can be achieved, for example, by infiltration of the groove with filler with a suitable refractive index. By varying the optical thicknesses of the defects simultaneously, the resonance (or channel) peak positions, *λ*_*c*_, and the *Q* factor of the coupled resonances obtained can be continuously tuned within the stop band (SB) [5,18,19]. For this purpose, the LC is a promising candidate material, with its capability of continuous reverse tuning of the refractive index over a wide range of values by the application of a voltage, *V*[20]. In this study, we use the commercially available nematic LC E7 available by Merck KGaA, Darmstadt, Germany, which demonstrates a high anisotropy of the refractive index $\left(\Delta n=1.7472\ldots 1.5217\right)$under applied voltages, *V* = 1.5…15 V [11-14]. For clarity, we consider an initial switching position that can achieve the same intermediate orientation of the LC molecules in all cavities. That is, it produces the average refractive index $n_{\text{LC}}=\left(1.7472+1.5217\right)/2=1.63$ at a switching voltage of approximately 10 V.

All modelled results are obtained using numerical simulations based on a transfer matrix method (TMM) [21]. The optimal design parameters for the LC-coupled microcavities, *d*_Si_*d*_air_ and *d*_def_ *= d*_LC_, which give the highest *Q* factor and the narrowest equal wavelength spacings between the channels, *λ*_*s*_, are determined using a graphical engineering approach, based on a Gap Map (GM) presentation [22]. The methodology of this approach is quite general and is described in detail in references [5,15,20]. In brief, optimal design parameters are determined by certain range of filling fractions, $\Delta f_{\text{Si}}=d_{\text{Si}}/a$, that correspond to the central part of the resonance lines within the SB on the GM and demonstrate the same improved optical characteristics, *λ*_*s*_ and *Q.* Depending on the order of the SB and the resonances within it, this Δ *f*_Si_ range varies. For example, for the triple-channel PhC device considered here (Figure 1), the filling fractions ranges are $\Delta f_{\text{Si}}=\text{0.15…0.25}$ (for the first order SBs), $\Delta f_{\text{Si}}=0.35\ldots 0.45$ (for the second order SBs) and $\Delta f_{\text{Si}}=0.47\ldots 0.57$ (for the third order SBs). Table 1 gives the TMM-simulated optimal design parameters, together with the optical characteristics of the sample triple-channel device [5].

**Table 1:** Optimal design parameters and channel characteristics of the first, second, third order SBs of triple-cavity PhC

***m***	***a = d***_**Si**_ **+** ***d***_**air**_**(nm)**	***f***_**Si**_	***d***_**Si**_**(nm)**	***λ***_***c*1**_**(nm)**	***λ***_***c*2**_**(nm)**	***λ***_***c*3**_**(nm)**	***λ***_***s***_ ***= λ***_**s12**_ **=** ***λ***_***s*23**_**(nm)**	***Q***	***I***_**1,2,3**_**(%)**
1	1,400	0.23	325	3,720	3,875	4,025	153.3	130	100
2	900	0.4	360	1,670	1,713	1,756	43	380	100
3	900	0.54	486	1,363	1,383	1,403	20	460	100

## Results and discussion

**Figure 2:**
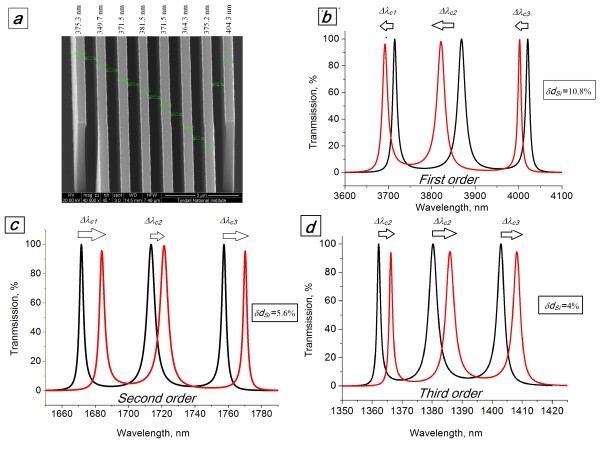
**SEM image and transmission spectra of the ideal and fabricated devices.** ( **a**) SEM image of the fabricated device with the thicknesses of the Si walls denoted by numbers. Transmission spectra of the predicted ideal triple-defect device (black line) and the fabricated device (red line) operating within ( **b**) the first order SB with *d*_Si_ = 325 nm, (**c**) the second order SB with *d*_Si_ = 360 nm and (**d**) the third order SB with *d*_Si_ = 486 nm. The structural deviations for the fabricated device are: (b) *± δ d*_Si_ *=* 10.8% (35 nm), (c) 5.6% (20 nm) and (d) 4% (20 nm). The refractive index of allcavities is $n_{\text{def}}^{\left(2,4,6\right)}=1.63$.

Optimal parameters taken from Table 1 were used for the fabrication of integrated triple-channel devices on a <100 > *p*-type silicon-on-insulator (SOI) wafer with a device layer thickness of 4.5 μm. The fabrication process involved used e-beam lithography to define the patterns followed by plasma etching down to the buried oxide layer. Scanning electron microscopy (SEM) investigation of the devices fabricated revealed the presence of a random deviation of the Si wall thicknesses, *δd*_Si_, of up to 10.8% of the target thickness. In Figure 2a, this deviation is demonstrated on the SEM image of the structure with *a* = 900 nm, which operates within the second order SB ( *m* = 2, Table 1). This thickness deviation, *δd*_Si_, directly influences the optical thicknesses of the cavities, $n_{\text{def}}^{\left(i\right)}\;d_{\text{air}}=n_{\text{def}}^{\left(i\right)}\;\left(a-d_{\text{Si}}\pm \delta d_{\text{Si}}\right)$, and also the optical thicknesses of the spacings between the cavities (or optical thickness of the air grooves),$n_{\text{air}}\;d_{\text{air}}=n_{\text{air}}(d_{\text{air}}\pm \delta d_{\text{Si}})$. Variation of the geometrical parameters of a coupled-cavity device results in random shifts, splitting and attenuation of each of the cavity resonances [14,23-25].

Simulated transmission spectra for the devices originally designed and fabricated are demonstrated in Figure 2b,c,d. Although the attenuation of the maximum transmission intensities is only about 3% to 5% resulting in a negligible reduction in the parameter *Q*, the precise wavelength position of each channel, λ_c_, was affected significantly. All of the channels are unequally blue/red-shifted by approximately 0.3% to 1.2% from the originally designed operational wavelengths, which is more than the target channel bandwidth (Figure 2b,c,d, red lines). A summary of the TMM analysis for the three fabricated structures, operating within the first three SBs along with data on the SEM registered thickness deviations, *±δd*_Si_, and the corresponding random shifts of the resonant peak wavelengths, ±Δ*λ*_*c*1,2,3_, are reported in Table 2.

**Table 2:** Deviation of the Si wall thicknesses and channel characteristics for the fabricated triple-cavity PhC

***m***	***d***_**Si**_ **+** ***d***_**air**_**(nm)**	***d***_**Si**_ ***± δ d***_**Si**_**(nm)**	**±Δ*λ***_***c*1**_**(nm)**	**±Δ*λ***_***c*2**_**(nm)**	**±Δ*λ***_***c*3**_**(nm)**	***λ***_***s***_ **± Δ*λ***_***s*12**_**(nm)*λ***_***s***_ **± Δ*λ***_***s*23**_**(nm)**
1	1,400	325 *±* 35	−22	−46.8	−18.5	153.3 − 24.3
		(***±***10.8 %)				153.3 + 27.7
2	900	360 *±* 20	+12.5	+7.7	+12.5	43 − 6
		(***±***5.6 %)				43 + 6
3	900	486 *±* 20	+4	+5.6	+5.3	20 − 1
						20 + 2
		(***±***4 %)				

The simplest way to compensate for these errors, post-fabrication (*d*_air_ ± *δd*_Si_), is to adjust the refractive indices of the coupled cavities individually to their optimised values, $n_{\text{def}}^{\left(2,4,6\right)}\pm \delta \;n_{\text{def}}^{\left(2,4,6\right)}$. This can be achieved by fabrication of the device on SOI platform and by applying a different voltage to each individual cavity. Then, by increasing or decreasing the applied voltage by Δ*V* in the various cavities, different variations in the LC refractive indices can be obtained, $n_{\text{def}}^{\left(2,4,6\right)}=n_{\text{LC}}\pm \Delta n$. For the example of a triple-cavity PhC, we focus on the two most obvious relationships between $n_{\text{def}}^{\left(2\right)}\text{,}\;n_{\text{def}}^{\left(4\right)}\text{ and }\;n_{\text{def}}^{\left(6\right)}$_,_ allowing precise manipulation of the central channel position, *λ*_*c*2,_ or the edge-channel positions, *λ*_*c*1_ and *λ*_3_.

**Figure 3:**
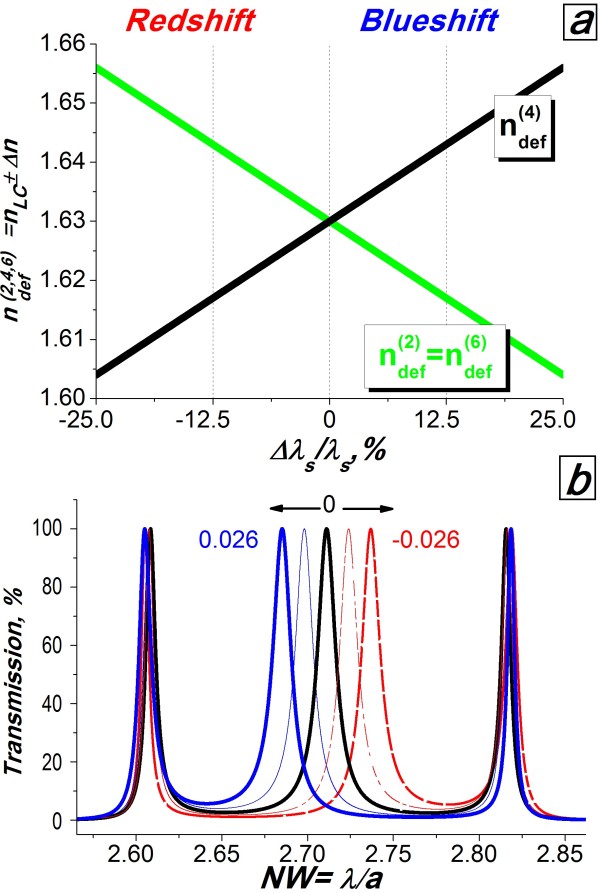
**Cavity refractive indices versus relative channel shift and continuous tuning of the central channel.** ( **a**) The cavity refractive indices, $n_{\text{def}}^{\left(2,4,6\right)}$, versus relative channel position shift normalised to the original channel spacing. (**b**) Continuous tuning of the central channel for *∆n* = −0.026 (red dashed line), *∆n* = −0.013 (red dashed dotted line), *∆n* = 0 (black line), *∆n* = 0.013 (thin blue line) and *∆n* = 0.026 (thick blue line).

Let us gradually increase the refractive index of the central cavity, $n_{\text{def}}^{\left(4\right)}=n_{\text{LC}}+\Delta n$, by *Δn* = 0.001, while decreasing the refractive indices of the two edge cavities, $n_{\text{def}}^{\left(2\right)}=n_{\text{def}}^{\left(6\right)}=n_{\text{LC}}+\Delta n$ by the same value of Δ*n* (Figure 3a). In this case, the central cavity has the strongest coupling, $n_{\text{def}}^{\left(2,6\right)}=n_{\text{def}}^{\left(2,6\right)}+2\;\Delta n$, and acts as the primary cavity in the system, while the edge cavities represent variable mirrors on both sides of the central cavity. At the same time, the overall optical thickness of the system $n_{\text{def}}^{\left(2,4,6\right)}\;d_{\text{air}}=\lambda_{m}/2$ decreases which leads to a blue shift of its resonance peaks [15]. Let us monitor the positions of the triple resonances during these refractive index variations. We use normalised wavelength units, NW = *λ/a*, and therefore, the results obtained can be applied to a variety of nano- and micro- structures, operating over a wide wavelength range. In Figure 3b, we demonstrate transmission spectra for *Δn* = 0.013 (thin blue line) and *Δn* = 0.026 (thick blue line). The central channel is blue-shifted, while the edge resonances have retained their positions. For the example considered, the maximum refractive index difference, 2 *Δn* = 0.052 (which is only 24% of the birefringence value for the LC E7, Δ *n*_E7_ *=* 0.2255), allows us to achieve a maximum relative independent shift of the central channel, Δ *λ*_*s*_, of up to 25% of the channel spacing, *λ*_*s*_. However, further increases in the overall difference, 2*Δn*, will break the symmetry of the system and initiate a coupling effect between all of the cavities, resulting in a shift of all the resonances simultaneously.

The opposite manipulation of the refractive indices, i.e. reducing $n_{\text{def}}^{\left(4\right)}=n_{\text{LC}}-\Delta n$ while increasing $n_{\text{def}}^{\left(2\right)}=n_{\text{def}}^{\left(6\right)}=n_{\text{LC}}+\Delta n$, results in an individual red shift of the central channel (Figure 3b, red dashed dotted and dashed lines). Note that the maximum intensity of *T* = 100% and the initial bandwidths are unaffected for all switching positions of the triple-channel system.

**Figure 4:**
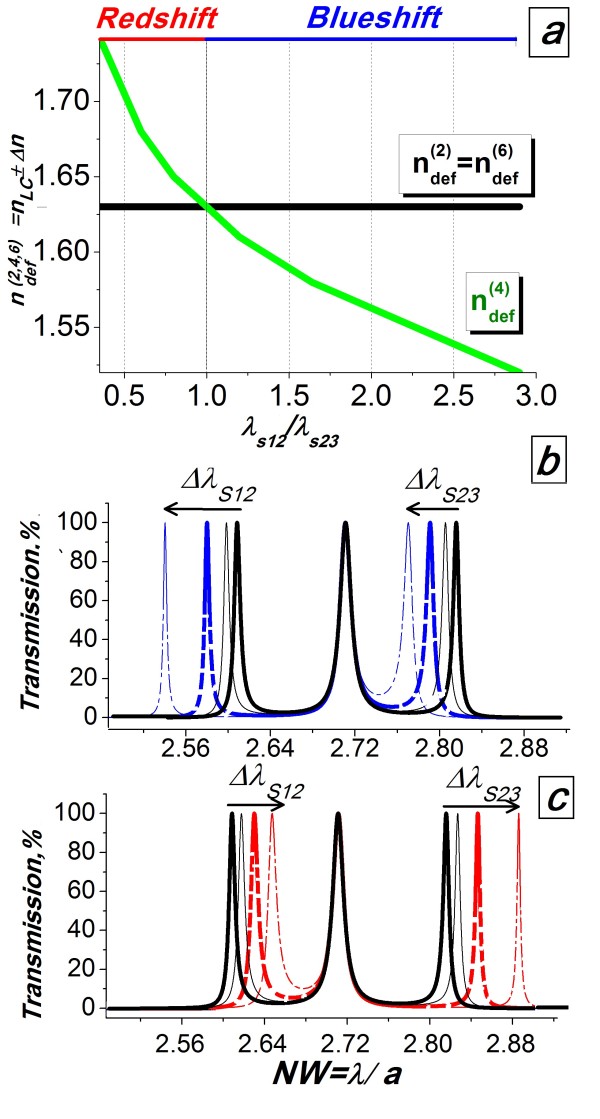
**The cavity refractive indices versus relative channel spacings and continuous tuning of the edge channels.** ( **a**) The cavity refractive indices $n_{\text{def}}^{\left(2,4,6\right)}$ versus the ratio of the channel spacings, *λ*_*s*12_/*λ*_*s*23_. (**b**) Continuous blue shift of the edge channels with a linear decrease in the central cavity refractive index, $n_{\text{def}}^{\left(4\right)}\text{,}$ by *∆n* = 0.02 (thin black line), *∆n* = 0.052 (dashed blue line) and *∆n* = 0.11 (dashed dotted blue line). The triple transmission channels, for *∆n* = 0, are also demonstrated (thick black line). ( **c**) Continuous red shift of the edge transmission channels with a linear increase in the central cavity refractive index, $n_{\text{def}}^{\left(4\right)}\text{,}$ by *∆n* = 0.02 (thin black line), *∆n* = 0.052 (red dashed line) and *∆n* = 0.11 (red dashed dotted line).

We now consider the effect of varying the refractive index of the central cavity only in $n_{\text{def}}^{\left(2\right)}=n_{\text{def}}^{\left(6\right)}=n_{\text{LC}}=1.63.$The refractive indices of the edge cavities are considered to be fixed at the initial value, $n_{\text{def}}^{\left(2\right)}=n_{\text{def}}^{\left(6\right)}=n_{\text{LC}}=1.63$ (Figure 4a). In this case, the central cavity plays the role of a variable intermediate mirror between two strongly coupled edge cavities. The decrease by *Δn* of the overall optical thickness of the system $n_{\text{def}}^{\left(2,4,6\right)}d_{\text{air}}=\lambda_{m}/2$ results in a blue shift of the edge channels, while the central channel retains its wavelength position. This is clearly demonstrated in Figure 4b for *Δn* = 0, 0.02, 0.052 and 0.11 for the structure under consideration. The increase of the channel spacing, *λ*_*s*_ *+ Δλ*_s_, results in a decrease of the channel spacing between the central channel and the right edge channel, *λ*_*s2,3*_ = *λ*_*s*_ *− Δλ*_*s*_, lowering the out-of-band reflection between them.

As in the previous model, the overall increase in the refractive index, *Δn* up to 0.052, for the example considered, leads to a linear change in the channel spacings of up to 25% or *λ*_*s*12_/*λ*_*s*23_ = 1.5 (Figure 4a, b). Although the out-of-band reflection between the central and right edge channel is decreased, it still reaches 95%. Further increases in *Δn* up to the limiting case of *Δn =* 0.11, will result in a rapidly growing difference between channel spacings, *λ*_*s*12_/*λ*_*s*23_ of up to 2.7 with a decrease of the out-of-band reflection between the central and left-edge channel to 90% (Figure 4b, blue dashed dotted line).

For significantly higher values of *Δn* > > 0.11 than those considered in this paper, the central cavity acts as a single cavity, independent of the edge cavities. The left-edge channel will be shifted out of the SB, and the right edge channel will be merged with the central channel, thus changing the resonator mode from triple mode to single mode. Obviously, the opposite manipulation of the refractive indices, i.e. increasing the $n_{\text{def}}^{\left(4\right)}=n_{\text{LC}}+\Delta n$ and fixed $n_{\text{def}}^{\left(2\right)}$ = $n_{\text{def}}^{\left(6\right)}=n_{\text{LC}}$, results in the individual red shift of the edge channels with the same Δ*λ*_*s*_ value (Figure 4c). Again, as for the central tuning combination, the maximum intensity of *T* = 100% is not affected for all switching positions of the triple-channel system.

**Figure 5:**
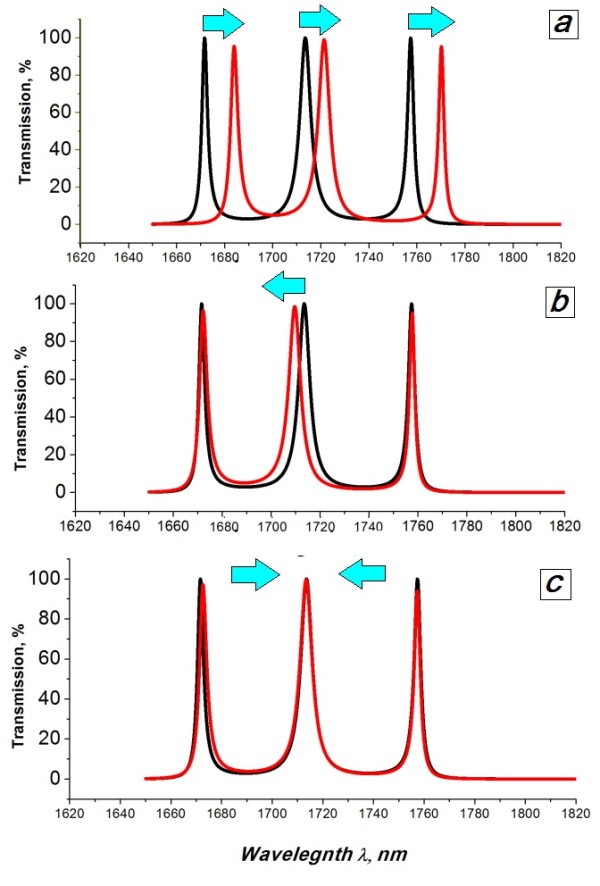
**Precise adjustment of the channel wavelength positions of the fabricated device to the originally designed**. Transmission spectra of the originally designed triple-defect device operating within the second SB ( *m* = 2, Table 1) (black line) and of the fabricated device with structural deviations of *± δ d*_Si_ *=* 20 nm = 5.6% ( *m* = 2, Table 2) (red line). (**a**) The refractive index of all cavities $n_{\text{def}}^{\left(2,4,6\right)}=1.63$. (**b**) Precise adjustment of the edge channel wavelength positions of the fabricated device to match those in the original design by decreasing the refractive index to $n_{\text{def}}^{\left(2,4,6\right)}=1.60$. (**c**) Final adjustment of the central channel with $n_{\text{def}}^{\left(4\right)}-∆n=1.60-0.01$ and $n_{\text{def}}^{\left(4\right)}-∆n=1.60-0.01$.

The tuning approach demonstrated here can be applied to the real devices (Table 2) as previously considered, allowing their actual characteristics to approach those in the original design (Table 1). In Figure 5, this is demonstrated for a sample fabricated structure with *m* = 2 (Figure 2a). First, we reduce the refractive index of all cavities, $n_{\text{def}}^{\left(2,4,6\right)}=n_{\text{LC}}-\Delta n$, in order to blue shift all three channels. The edge channels are precisely adjusted to the ideal positions when $n_{\text{def}}^{\left(2,4,6\right)}=1.63-0.03=1.60$ (Figure 5b, red line)*.* Now, we fine tune the central channel by increasing the refractive index of the edge cavities $n_{\text{def}}^{\left(2,6\right)}=1.61+\Delta n=1.6+0.01=1.61$ and reducing the refractive index of the central cavity $n_{\text{def}}^{\left(4\right)}=1.59$ (Figure 5c). The system is now operating at the nominal values in the original design.

## Conclusion

To summarise, an approach based on the individual tunability of multi-channel filters has been proposed for the compensation of optical parameter deviations caused by structural fluctuations. Electro-optical tuning of individual central (or edge) channels was demonstrated by up to 25% of the channel spacings, which is sufficient for the optimization of devices with fabrication tolerances of up to 20%. The approach suggested here can be utilised for the optimization of multi-channel silicon devices over a wide infrared range.

## Abbreviations

LC, liquid crystal; PhC, photonic crystal; SB, stop band; SEM, scanning electron microscopy; TMM, transfer matrix method.

## Competing interests

The authors declare that they have no competing interests.

## Authors’ contributions

AB is a principle investigator of the project and carried out the simulations, modelling and fabrication of the devices under supervision of TS-P. AB and TS-P designed the manuscript layout. VA-T, KB and TS-P participated in the drafting of the manuscript and helped with the analyses and interpretation of the data. All authors read and approved the final manuscript.

## Authors’ information

AB is a final year PhD student doing research under supervision of TS-P, professor and director of Microelectronics Technology Group in Trinity College Dublin, Ireland. VA-T is a senior researcher at Ioffe Physical Technical Institute, Russia, and KB is a lecturer at Dublin Institute of Technology, Ireland.

## References

[B1] Reed GT Silicon Photonics: The State of the Art 2008 Wiley, West Sussex

[B2] Wehrspohn RB Kitzerow HS Busch K Nanophotonic Materials: Photonic Crystals, Plasmonics, and Metamaterials 2008 Weinheim, Wiley-VCH

[B3] Pavesi L Lockwood DJ: Silicon Photonics 2004 Springer, Heidelberg

[B4] Postnikov S Hector S Garza C Peters R Ivin V Critical dimension control in optical lithography Microelectron Eng 2003 69 452 458 10.1016/S0167-9317(03)00334-4

[B5] Baldycheva A Tolmachev VA Perova TS Laurent V, Seppo KH, Pavesi L, Pelli S Fine tunable multi-cavity Si photonic crystal filter Proceedings of SPIE2012, 8431, April 16 2012 2012 SPIE Digital Library, Brussels 84310H 84310H-13

[B6] Ghulinyan M Oton CJ Gaburro Z Bettotti P Pavesi L Porous silicon free-standing coupled microcavities Appl Phys Lett 2003 82 1550 1552 10.1063/1.1559949

[B7] Mancinelli M Guider R Bettotti P Masi M Vanacharla MR Pavesi L Coupled-resonator-induced-transparency concept for wavelength routing applications Opt Express 2011 19 12227 12240 10.1364/OE.19.012227 21716460

[B8] Atabaki AH Momeni B Eftekhar AA Hosseini ES Yegnanarayanan S Adibi A Tuning of resonance-spacing in a traveling-wave resonator device Opt Express 2010 18 9447 9455 10.1364/OE.18.009447 20588791

[B9] Monat C Domachuk P Eggleton BJ Integrated optofluidics: a new river of light Nat Photon 2007 1 106 114 10.1038/nphoton.2006.96

[B10] Alboon SA Lindquist RG Flat top liquid crystal tunable filter using coupled Fabry-Perot cavities Opt Express 2008 16 231 236 10.1364/OE.16.000231 18521153

[B11] Weiss S Ouyang H Zhang J Fauchet P Electrical and thermal modulation of silicon photonic bandgap microcavities containing liquid crystals Opt Express 2005 13 1090 1097 10.1364/OPEX.13.001090 19494976

[B12] Tolmachev VA Melnikov VA Baldycheva AV Perova TS Fedulova GI Miguez HR, Romanov SG, Andreani LC, Seassal C Design, fabrication, and optical characterization of Fabry-Prot tunable resonator based on microstructured Si and liquid crystal Proceedings of SPIE 2010, April 12 2010 2010 SPIE Digital Library, Brussels, Belgium 771320 771312

[B13] Pucker G Mezzetti A Crivellari M Bellutti P Lui A Daldosso N Pavesi L Silicon-based near-infrared tunable filters filled with positive or negative dielectric anisotropic liquid crystals J Appl Phys 2004 95 767 769 10.1063/1.1630692

[B14] Cos J Ferré-Borrull J Pallarès J Marsal LF Double-cavity Fabry–Pérot tunable equalizer based on 1D photonic crystals Int J Numerical Model: Electron Netw, Devices and Fields 2010 23 400 410

[B15] Tolmachev VA Perova TS Baldycheva AV Kubby JA, Reed GT Transformation of one-dimensional silicon photonic crystal into Fabry-Perot resonator Proceedings of SPIE 2011, 7943 January 22 2011 2011 SPIE Digital Library, San Francisco 79430E 79410

[B16] Gaponenko SV Introduction to Nanophotonics 2010 Cambridge University Press, Cambridge

[B17] Pavesi L Gaponenko SV Negro LD: Towards the First Silicon Laser 2003 Kluwer Academic Publishers, Dordretcht

[B18] Wang Z Peng RW Qiu F Huang XQ Wang M Hu A Jiang SS Feng D Selectable-frequency and tunable-Q perfect transmissions of electromagnetic waves in dielectric heterostructures Appl Phys Lett 2004 84 3969 3971 10.1063/1.1748848

[B19] Mudachathi R Nair P Low-voltage widely tunable photonic crystal channel drop filter in SOI wafer Microelectromechanical Syst, J 2012 21 190 197

[B20] Tolmachev VA Melnikov VA Baldycheva АV Berwick K Perova TS Electrically tunable Fabry-Perot resonator based on microstructured Si containing liquid crystal Prog In Electromagnetics Res 2012 122 293 309

[B21] Azzam RMA Bashara NM Ellipsometry and polarized light 1987 North-Holland, Elsevier Science Publisher

[B22] Joannopoulos JD Photonic Crystals: Molding The Flow Of Light 2008 Princeton, Princeton University Press

[B23] Pavesi L Panzarini G Andreani LC All-porous silicon-coupled microcavities: Experiment versus theory Phys Rev B 1998 58 15794 15800 10.1103/PhysRevB.58.15794

[B24] Marcuse D Coupled mode theory of optical resonant cavities Quantum Electron, IEEE J 1985 21 1819 1826 10.1109/JQE.1985.1072590

[B25] Stanley RP Houdre R Oesterle U Ilegems M Weisbuch C Coupled semiconductor microcavities Appl Phys Lett 1994 65 2093 2095 10.1063/1.112803

